# 1903

**Published:** 1904-01-02

**Authors:** 


### HOSPITAL CLINICS AND MEDICAL PROGRESS.

### 1903.

In looking back upon the last twelve months and reviewing the work that has been accomplished during that time towards the advancement of medicine and surgery, we have the pleasing retrospect of a year of substantial progress in almost every department. It is also satisfactory to perceive a growing interest taken by the general public in the wider medical problems and issues of the day, as shown by the frequency with which such matters are discussed, and the prominence which is given to them, by our lay contemporaries. This is indeed fortunate, for what is now most required for a continuance of the successful struggle against human ailments is money. Funds are wanted to effect improvements in medical education, to furnish, equip, and support institutions for research, and to establish our voluntary hospitals both in London and in the provinces on a footing which will enable the utmost advantage to be taken of modern knowledge in the treatment of the sick poor, and which will give the best opportunities for learning to those who are engaged in the study of medicine. There is good reason for believing that the necessary support will be forthcoming, on account both of the generous manner in which the public are responding to the large appeals which are now before them and the increasing prosperity and popularity of the central hospital funds.

### Hospital Problems.

The chief matters with which the authorities of the voluntary hospitals of London have had to deal are the rebuilding and enlargement of the institutions under their care in order to keep pace with the modern advance of medical knowledge as well as to meet the requirements of ever-increasing numbers of people seeking medical relief, and the consideration of hospital sites in connection with the redistribution of the population which has been such a remarkable feature of the last thirty or forty years. Another point which has arisen lately is the question of whether urban hospitals are suitable for the reception of patients who are suffering from certain chronic diseases. It has been shown by Mr. Tubby that children affected with tuberculosis of the bones, joints, or lymphatic glands as a rule do much better when they are treated out of London than when they are treated in one of the central hospitals. A committee appointed by the Society for the Study of Children's Diseases to inquire into the matter has reported in favour of Mr. Tubby's view that the urban treatment of surgical tuberculosis is not justifiable. Further investigation may be expected to prove that tuberculosis is not the only disease which is better treated in the country where there is plenty of fresh air. Here then are some momentous matters to be sounded. More than one of the great general hospitals finds itself on too small a site and yet unable to expand because of the value of surrounding land; the resident population is rapidly departing to the suburban districts, while modern notions of medical treatment insist with ever-increasing force upon the value of fresh air, which cannot be got in the centre of London. Certainly in the suburbs there is better air, land is more easily obtained, and there are the people who need the help of a hospital. These considerations have led the governors of King's College Hospital to decide upon removing that institution from the cramped and unsuitable site where it now stands to a new position in South London where hospital accommodation is very deficient. The Hon. W. F. D. Smith, M.P., has generously placed a site of about twelve acres in Camberwell at the disposal of the authorities, while their appeal for a sum of £300,000 for the purposes of rebuilding is already meeting with a generous response. It may be remembered that King Edward's Hospital Fund for London has been able to distribute during the year £100,000, while with the help of another noble contribution by Mr. George Herring, the Metropolitan Hospital Sunday Fund has distributed £58,386. The Hospital Saturday Fund exceeds last year's total.

The League of Mercy has still further widened its field of activity, and has been able to place no less a sum than £10,000 in the hands of King Edward's Hospital Fund for London. So much for the Hospitals.

### Medicine and Surgery.

In medical work perhaps the most notable advance has been made in tropical diseases, while cancer research, the prevention and treatment of pulmonary tuberculosis, and the prevention of infantile diarrhœa, have received especial attention. In surgery progress has been chiefly concerned with the technical details of various procedures and the eternal question of whether certain conditions require operation or whether they are best left alone, for a time at any rate. This question has been raised more prominently perhaps with regard to uterine fibroids and cancer of the stomach, and for certain of the complications of simple gastric ulcer. Although these disputatious matters are as far from settlement as possible, and denote no particular advance in surgery, yet they have led to the accumulation of a large number of exceedingly useful data, which will serve the purpose of guiding the surgeon in individual cases which come under his care.

### Tropical Diseases.

The most important event of the year was, no doubt, the discovery of the cause of sleeping sickness. Dr. Castellani, in a communication read before the Royal Society on May 14th, reported that he had observed trypanosomes in several instances in the cerebro-spinal fluid removed by lumbar puncture from patients who were suffering from sleeping sickness in Uganda. Previous to this he had noticed trypanosomes in the blood of a certain number of cases of sleeping sickness, but considered their presence as probably fortuitous. However, on reporting his observations to Col. Bruce, one of the members of the Sleeping Sickness Commission of the Royal Society, the latter was struck with the possibility of the trypanosomes being the cause of the disease, and urged Dr. Castellani to follow up the clue, the result being to establish beyond doubt a causal relation between the presence of trypanosomes in the cerebro-spinal fluid and the symptoms of sleeping sickness. The next step was to discover, if possible, the means through which infection was conveyed. There were several indications that some blood-sucking insect might be the carrier of the trypanosomes. Thus the distribution of sleeping sickness, the nature of the parasite, which precluded the possibility of convection by air or contaminated food, and the analogy between sleeping sickness and *nagana*, the fly disease of South Africa so deadly to horses, which was known to be due to a similar parasite conveyed by a species of tsetse fly (*Glossina pallidipes*), led to an investigation which resulted first of all in a suspicion and later a certainty that the trypanosome of sleeping sickness is conveyed to human beings by a species of tsetse fly (*Glossina palpalis*). Now that the cause of this destructive disease is known as well as the manner in which it is communicated to man it is justifiable to look forward to a reduction of its prevalence and ultimately its total elimination from the diseases of mankind. In many other branches of tropical medicine a great deal of good work has been done. The warfare against malaria has been rewarded with more or less success in most parts of the world where this scourge is rife. Indeed the results attained in this direction are sufficient to be a very happy augury for the future of many climates where malaria has hitherto prevailed. Considerable attention is at present being devoted to beriberi, and it is to be hoped that before long the pathology of this affection also will be unravelled.

### Cancer Research.

The past year has witnessed the commencement of an organised and systematic endeavour to fathom the mysteries of malignant disease. This is now being carried out under the direction of the Cancer Research Fund, and the report of the first year's work was presented to the General Committee on July 30th. Although no great advance has been made, yet many important facts have been collected and the ground well prepared for future investigation. There is one noteworthy statement in the report and that is relative to the value of $x$-rays and other forms of electric treatment in cancer of which so many conflicting opinions have been circulated. The position at present is that the $x$-rays are of value in the treatment of rodent ulcer. With regard to other forms of malignant disease it is stated that no results have been brought forward which establish the efficiency as a curative agent in cases of either sarcoma or carcinoma of any form of electrical treatment. So far as it goes such a statement is mainly of value as a check to those who would too hastily travel along an insufficiently explored track to the ultimate grief of their patients and disappointment of themselves. With regard to the pathology of cancer we seem to be in much the same position as before. Mr. Henry Morris, chairman of the Cancer Investigation Committee of the Middlesex Hospital, and treasurer of the Cancer Research Fund, stated in his Bradshaw Lecture on December 9th that the parasitic theory of cancer had been well put to the test and found wanting, and that Cohnheim's theory at present offered the most promising field for research. Cohnheim, it will be remembered, attributes the origin of carcinoma to the proliferation of embryonic epithelial cells which during fœtal life have been cut off from their proper connections and remained in an undeveloped state. In contrast with this statement by Mr. Morris, other pathologists are urging more strongly than ever the parasitic theory, and one, Dr. Schmidt, of Cologne, even goes so far as to say that he can isolate the parasite and cultivate it in vitro, and produce by artificial means a serum, which, if not actually curative, produces when injected subcutaneously a marked reaction in cancerous growths, and causes the cancer cells to die and to be replaced by apparently normal scar tissue. One naturally enough feels a good deal of scepticism on the point in view of previous disappointments; but as Schmidt's serum is now being put to the test in London the matter should soon be placed in a clearer light. We are informed that up to the present the cases treated in London have not shown much promise of improvement. So far as the treatment of cancer is concerned, progress has chiefly been made in the direction of early diagnosis and improved operative technique.

### Electro-therapeutics.

The most important innovation has been made by M. and Mde. Curie in the discovery of radium. Owing to the expense of this substance and the short time that has elapsed since its discovery was announced, we are not yet in a position to estimate its possible uses in medicine, but sufficient is known already to affirm that in some instances, at any rate, of small rodent ulcers and lupus the radium rays exert a curative action. If they stand the test of time, and if the supply of radium becomes more plentiful, there is reason to hope it may become a useful therapeutic agent in the hands of the general practitioner.

### Prevention and Treatment of Tuberculosis.

That the time has come for taking some more energetic measures to check the prevalence of this plague scarcely anyone is likely to deny. Such steps as have been taken during the past year have mainly been in the direction of educating the general public so that they may sufficiently understand the importance of the question and the value of any measures it may be proposed to adopt. The London County Council has indeed taken a definite step in the right direction by means of a by-law which forbids spitting, under penalty of a fine, in any of the Council's public conveyances. The gospel of fresh air is being carried even into the homes of the most squalid, and together with it is being taught the infectiousness of pulmonary tuberculosis. All these efforts bear the promise of fruit in the near future.

A good deal of energy has been devoted to the erection of much-needed sanatoria for the treatment of consumptives throughout the country. On November 3rd the King laid the foundation stone of the open-air sanatorium which is to bear his name at

Midhurst. There is so much need for institutions for the treatment of consumptives that it may be doubted if the community will be able to supply the money required for the erection of enough large and solid buildings for the purpose. At the same time it has been shown that patients with pulmonary tuberculosis get on remarkably well in wooden or otherwise constructed huts, and even in tents. As such structures can be erected at one-tenth of the cost of ordinary sanatorium buildings, it is to be hoped that they will take the place of the latter in future plans for sanatoria, and so effect a saving of public money and at the same time provide ample accommodation for the consumptives.

### Prevention of Infantile Diarrhœa.

The mortality among infants has undergone no decrease in spite of all the great strides made during the last forty years in England. This mortality is mainly due to errors in feeding, and is mostly confined to babies who are not breast-fed. Modern conditions of life have increased the proportion of artificially-fed babies. During the last twelve months this question has attracted a good deal of attention, and several papers have appeared giving an account of the steps that have been taken to rectify the evil in France and other parts of the Continent, and also in a few places in this country, among which St. Helens and Battersea may be quoted for examples. The general procedure, which has been shown to be very successful, is the supply of sterilized milk on an extensive scale, and consequently at small cost to every mother who applies for it daily. Sufficiently large statistics are now to hand to show that the babies fed on this system do not die in anything like the same numbers as artificially fed infants do whose daily food is prepared at home. Doubtless the babies' milk-shop will be in time an essential feature of every quarter where the population is dense.

### Femoral Hernia.

The treatment by operation of femoral hernia has always been unsatisfactory, owing to the difficulty of closing the abdominal opening of the femoral canal. The various methods of doing this, such for example as by suturing Poupart's ligament to the pectineal fascia, or by fastening the ligament to the pubic bone by means of a staple, have their objections. Sir Victor Horsley has suggested a new plan which he believes to be successful, and which he has carried out in several cases. His method is to make an incision above Poupart's ligament and chisel off a scale of bone from the upper and back surface of the os pubis leaving it attached only by the periosteum adjacent to the femoral ring. The bone is then levered over so as to cover the femoral ring like a lid, and is sutured in this position by a single suture.

### Facial Paralysis.

In an interesting paper Messrs. C. A. Ballance, H. A. Ballance, and Dr. Stewart have called attention to the results which can be obtained by operation in cases of complete facial paralysis which have not proved amenable to time and medical treatment. They give the notes on seven cases. In six of these a junction was effected between the distal end of the facial nerve and the spinal accessory nerve, in the seventh a facio-hypoglossal anastomosis was performed. In five of these sufficient time had elapsed since the operation to judge of the result. In four of these patients the paralysis was due to chronic otitis media, while in the remaining one it followed fracture of the skull. The results were fairly uniform. The facial muscles recovered their contractility, but could only be brought into action in conjunction with the muscles supplied by the spinal accessory nerve, and conversely whenever the muscles supplied by the latter nerve were brought into action the facial muscles also contracted. In the case in which the facial nerve had been joined to the hypoglossal, sufficient time had not elapsed to observe the result. With regard to the length of time the facial paralysis has existed previous to operation, the authors consider that no interval of time is too long for attempted cure so long as any muscle fibres survive, as shown by response to galvanic stimulation.

### Birth Paralysis of the Upper Extremity.

Professor Robert Kennedy points out that the cause of this affection is an injury received during birth to the anterior divisions of the fifth and sixth cervical nerves at their point of junction. In cases which do not improve with milder measures, Dr. Kennedy recommends operation without undue delay. If no response can be got in the affected muscles by the faradic current by the time the child is two months old it is advisable, he thinks, to wait no longer, but to cut down upon the upper root of the cervical plexus, and if the nerve junction is found to be cicatricial, as was the case in all of his own cases, to excise the cicatrised portion and join up the divided ends.

### Internal Urethrotomy.

In dealing with a case of stricture Mr. Lockwood advises that the operation should be carried out with all the antiseptic arrangements of an abdominal operation. No instrument should be passed until the patient is anæsthetized. The pubes must be shaved, the skin of the abdomen scrupulously disinfected, and the field of operation isolated by sterilized towels. The stricture is then measured, and, if internal urethrotomy be possible, is divided from behind forwards by means of a special form of urethrotome devised by himself to pass through strictures of narrow calibre.

### Surgery of the Stomach.

The stomach has lately attracted a great deal of notice from surgeons. Among other conditions, that of congenital hypertrophic stenosis of the pylorus has come into prominence, a number of cases having been recorded by Mr. Clinton Dent and others, so that the condition does not appear to be so rare as it was formerly supposed to be. Other progress which has been made chiefly refers to earlier operation in cases of carcinoma and simple ulcer of the stomach.

### Honours to Members of the Medical Profession.

In connection with the Coronation Durbar at Delhi honours were conferred upon several members of the medical profession including the following :—

Surgeon-General William Roe Hooper, C.S.I., I.M.S. (retired), President of the Medical Board at the India Office, was created K.C.S.I.; Surgeon-General Benjamin Franklin, C.I.E., Director-General of the Indian Medical Service and Sanitary Commissioner with the Government of India, was created K.C.I.E.; Lieutenant-Colonel Gerald Bomford, M.D., I.M.S., Principal of the Medical College, Calcutta, and Major Alfred William Alcock, M.B., F.R.S., I.M.S., Superintendent of the Indian Museum were created C.I.E. The Kaisar-i-Hind medal for public service in India of the first class was conferred on Leiutenant-Colonel Robert William Steele Lyons, M.D., I.M.S., Superintendent of the Lunatic Asylum, Dharwar, and upon Major David Semple, M.D., R.A.M.C., Director of the Pasteur Institute, Kasauli. The honour of knighthood was conferred upon George Watt, M.B., C.M.Glasg., C.I.E., Reporter on Economic Products to the Government of India.

Among the King's birthday honours the names of several distinguished members of the medical profession were included. Dr. Patrick Manson was appointed to Knight Commandership of the Bath. The honour of knighthood was conferred upon Mr. Alfred Fripp, and Drs. Stephen Mackenzie, E. C. Perry and P. Heron Watson. Surgeon-General Colvin Colvin-Smith, C.B, I.M.S., and Surgeon Major-General John By Cole Reade, C.B., were advanced to K.C.B. Surgeon-General Evatt and Surgeon-General Scott Reid were nominated C.B., while the honour of C.M.G. was conferred on Mr. Allen Hastings Hanley, F.R.C.S.I., Senior Medical Officer of the Protectorate of Southern Nigeria.

Sir John Williams was invested by the King with the insignia of a Knight Commander of the Royal Victorian Order, while Dr. Friedrich Ilberg, M.V.O., Physician to the German Emperor, was promoted to be a Commander of the Order.

On the occasion of his Majesty's visit to Ireland the King bestowed the honour of knighthood on Dr. Arthur Vernon Macan, President of the Royal College of Physicians of Ireland, and Dr. Lambert Hepenstal Ormsby, President of the Royal College of Surgeons of Ireland.

In the second list of birthday honours was the name of Dr. Alan Reeve Manby, Surgeon-Apothecary to the King's household at Sandringham, who was created knight. Captain T. H. M. Clarke, M.B., R.A.M.C., D.S.O., was nominated a Companion of the Order of St. Michael and St. George for services in Crete. Dr. Robert Bell, F.R.S., was appointed a Companion of the Imperial Service Order.

